# Effect of Health Information Technologies on Cardiovascular Risk Factors among Patients with Diabetes

**DOI:** 10.1007/s11892-019-1152-3

**Published:** 2019-04-27

**Authors:** Yilin Yoshida, Suzanne A. Boren, Jesus Soares, Mihail Popescu, Stephen D. Nielson, Richelle J. Koopman, Diana R. Kennedy, Eduardo J. Simoes

**Affiliations:** 1 Department of Health Management and Informatics, School of Medicine, University of Missouri-Columbia, CE707 CS&E Bldg., One Hospital Drive, Columbia, MO 65212 USA; 2 Missouri Cancer Registry and Research Center, University of Missouri-Columbia, Columbia, MO USA; 3 Centers for Disease Control and Prevention, Division of High-Consequence Pathogens and Pathology, Prion and Public Health Office, Atlanta, GA USA; 4 Mercy Medical Center, Sioux City, IA USA; 5 Department of Family and Community Medicine, School of Medicine, University of Missouri-Columbia, Columbia, MO USA

**Keywords:** Health information technologies, Type 2 diabetes, Cardiovascular risk factor

## Abstract

**Purpose of Review:**

To identify a common effect of health information technologies (HIT) on the management of cardiovascular disease (CVD) risk factors among people with type 2 diabetes (T2D) across randomized control trials (RCT).

**Recent Findings:**

CVD is the most frequent cause of morbidity and mortality among patients with diabetes. HIT are effective in reducing HbA1c; however, their effect on cardiovascular risk factor management for patients with T2D has not been evaluated.

**Summary:**

We identified 21 eligible studies (23 estimates) with measurement of SBP, 20 (22 estimates) of DBP, 14 (17 estimates) of HDL, 14 (17 estimates) of LDL, 15 (18 estimates) of triglycerides, and 10 (12 estimates) of weight across databases. We found significant reductions in SBP, DBP, LDL, and TG, and a significant improvement in HDL associated with HIT. As adjuvants to standard diabetic treatment, HIT can be effective tools for improving CVD risk factors among patients with T2D, especially in those whose CVD risk factors are not at goal.

**Electronic supplementary material:**

The online version of this article (10.1007/s11892-019-1152-3) contains supplementary material, which is available to authorized users.

## Introduction

Cardiovascular disease (CVD) is recognized as the most frequent cause of morbidity and mortality in patients with diabetes, causing up to 70% of all deaths in this patient group [1•]. Type 2 diabetes (T2D) confers an approximate twofold elevation of CVD risk, equivalent to that of a previous myocardial infarction [2, 3]. Controlling CVD risk factors, such as hypertension, dyslipidemia, hypertriglyceridemia, and obesity, and targeting strategies to promote cardiovascular health are key in managing unfavorable microvascular and macrovascular outcomes and reducing CVD-related death in patients with diabetes.

Less than half of patients with diabetes who regularly visit their care provider meet recommended levels for blood pressure (BP) and lipids [4]. Innovative approaches are needed to improve cardiovascular risk management for this patient group. Health information technologies (HIT) include a broad category of technologies, electronic tools, applications, or systems that provide patient care, information, recommendations, and services for health management [5]. Emerging evidence has shown HIT’s role in enhancing chronic disease management [5, 6] via supporting provider decision-making (through electronic risk assessment, alerts, guidelines, formularies, and prescribing) and facilitating patient self-management (through risk communication, web portals, telemedicine, e-mailing, and secure messaging) [6]. In the context of cardiovascular care, HIT offer numerous benefits and have been associated with improvements in the measurement and monitoring of heart health, including risk factors such as blood pressure, arrhythmia, cholesterol, and weight, as well as the implementation of guideline-based decision support for providers [7]. With respects to glycemic control, our recent meta-analysis has demonstrated a significant reduction in HbA1c, both statistically and clinically, resulted from applied HIT [8••].

Existing systematic reviews and meta-analyses examining HIT’s effect in diabetes management often lack adherence to standard quantitative method [9, 10], overlook CVD risks [11, 12], or include insufficient sample size or limited CVD parameters for analysis [13, 14]. Because CVD is the major cause of death among T2D patients, the evaluation of RCTs studying the effect of HIT on diabetes management should focus not only on glycemic control but also on CVD risk management. We synthesized the findings of HIT’s effect on primary CVD risk factors among patients with T2D who were subjects in trials to evaluate the effect of HIT on T2D.

## Methods

### Information Sources and Search Strategy

We systematically searched Medline for eligible articles through December 2017, using combinations of the following MeSH (M) and textword (TW) search terms: (1) Diabetes Mellitus Type 2 (M), diabetes (TW), diabetes mellitus (M), prediabetic state (M), and prediabetes (TW), and (2) telemedicine (M), mHealth (TW), cell phone (M), cell phone$ (TW), mobile phone$ (TW), telehealth (TW), eHealth (TW), internet (M), ambulatory monitoring (M), and wearable$ (TW). Similar searches were conducted in Cumulative Index of Nursing and Allied Health Literature (CINAHL) and the Cochrane Library. We also used Google Scholar to identify additional studies not listed in the above-mentioned databases. We also performed supplementary searches using the reference lists of eligible articles and relevant systematic review and other review articles we encountered.

### Eligibility Criteria

Studies were deemed eligible if they were peer-reviewed RCTs containing methodology and results sections that studied the effect of HIT on T2D with specific measurements on both HbA1c and CVD risk factors. Studies were excluded if they only included patients with type 1 diabetes, did not include cardiovascular risk factor measurements, involved continuous glucose monitors, were feasibility trials, or were not written in English.

### Data Screening

A multistage screening process was used whereby search results were first pooled and duplicates were removed. Next, article abstracts were screened for apparent relevance, and then the article texts were reviewed to confirm eligibility status. Articles extracted from reference lists underwent an identical process.

### Data Extraction

Following the screening process, data from eligible articles were extracted independently by two researchers. A coding manual was used to maintain reliable practices. The coding manual specified study characteristics (percentage of patients with diabetes, basic demographic data, and geographic setting), intervention characteristics (mobile technology utilized, education provided in the intervention, intervention delivery personnel, equipment provided, intervention length), and clinical outcomes (systolic blood pressure (SBP), diastolic blood pressure (DBP), high-density lipoprotein (HDL), low-density lipoprotein (LDL), triglycerides (TG), and weight). We focused on LDL instead of total cholesterol because it has now largely replaced total cholesterol as the primary lipid measurement for evaluation of risk due to atherogenic lipoproteins [15]. We included HDL in the review for lipid control because several studies have shown that low HDL (defined as < 40 mg/dL in both sexes or < 40 mg/dL in men and < 50 mg/dL in women) is an independent risk factor for CVD in both people with or without diabetes [16–20]. For all outcomes, additional data were extracted concerning the intervention’s treatment effect compatible with meta-analysis. Discrepancies were unanimously resolved before final data entry.

### Methodological Quality Assessment

Two reviewers assessed the quality of each article using the Cochrane Collaboration risk of bias assessment tool [21]. Six domains of bias (i.e., selection, performance, detection, attrition, reporting, and other) are included in the tool and risk scored as low, high, or unclear [21]. For each study, we summed domain scores to determine an overall score with risk of bias gauged low, unclear, or high. Assessors discussed their assessment discrepancies to reach consensus. We assessed the risk of selective reporting or publication bias by visual inspection of a funnel plot and fail-safe N test [21].

### Quantitative Synthesis

RCTs containing methodology and results sections that studied the effect of HIT on CVD risk factors among patients with T2D were eligible for meta-analysis inclusion. We used the Comprehensive Meta-Analysis version 3 (CMA) [22] to calculate two effect size measures. First, we calculated the difference in means. Second, we calculated an effect size measure adjusted to bias attributed to the use of different populations across studies using a random effects model (Hedges’ g effect size) [23]. Missed SDs were imputed using the pooled SD from all the other studies in the same meta-analysis [24]. Heterogeneity of each model was assessed using Cochran’s Q and *I*^2^ statistics [23]. We considered heterogeneity to be greater than expected by chance alone if either the Cochran’s Q showed *P* < 0.05 or the *I*^2^ statistics was ≥ 50% [25]. Although the effect of HIT on CVD risk factors represents a mix of both HIT and standard diabetes care, including medication adherence and lifestyle modifications, in some reviewed trials, the effect of each was not clearly distinguished (i.e., treatment information, including medication and lifestyle therapies, in the control group was not specified, or standard care components were unclear in both intervention and control groups). For this reason, we repeated the overall synthesis analysis using data from the trials (SBP *n* = 6, DBP *n* = 5, LDL = 4, HDL *n* = 5, and TG *n* = 5) that compared outcomes between a combined HIT and standard care intervention group and standard care alone control group.

## Results

**Fig. 1:**
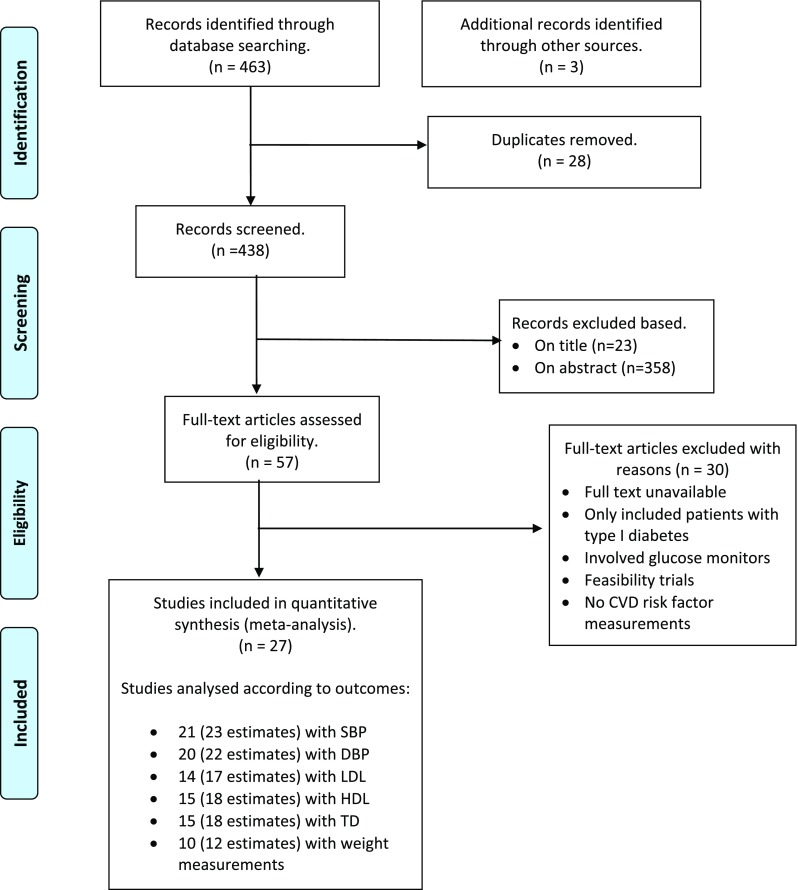
Article screening process (PRISMA 2018 flow diagram)

We identified 27 studies that have CVD risk factor measurements (Supplemental Table 1). Among these, 21 eligible studies (23 estimates) with measurement of SBP, 20 (22 estimates) of DBP, 14 (17 estimates) of HDL, 14 (17 estimates) of LDL, 15 (18 estimates) of TG, and 10 (12 estimates) of weight were identified and included in analyses (Fig. 1).

### Characteristics of Included Studies

The included studies were conducted in several different countries and regions: 6 in the USA [26–31], 9 in Europe [32–40, 41], 6 in South Korea [42–47], 2 in China [48, 49], 1 in Iran [50], 1 in Canada [51], 1 in India [52], and 1 in Japan [53]. The total participants were 3290, of which 1977 were randomized to intervention groups and 1313 to control groups. The majority of studies focused on T2D (22 out of 27, 81%); 3 (11%) with unclear information on diabetes type; 1 (4%) included both type 1 and type 2 diabetes. The mean age of participants ranged from 45 to 67 years old. Most studies had even gender distribution (85%). Three studies mainly focused on men (11%). One study had no information on gender (4%). Twenty (74%) utilized mobile phone-based applications as intervention tools. Of these, 7 were hybrid-interventions that primarily used mobile-phones to deliver treatments or services but also incorporated other applications, such as web-based applications in their programs. Three studies (11%) used web-based applications as major intervention components. Four studies (15%) used SMS/text. One study (4%) used video talks. Regarding control groups’ treatment, participants in the majority of studies (*n* = 17, 63%) received standard care and/or consultation from health care professionals. Control groups in 5 studies (19%) were engaged in diabetes self-management and/or education. Five studies (19%) had unclear information on treatment for control groups. With regard to intervention delivery personnel, 10 studies (37%) had a combination of medical care providers to deliver interventions. Four studies (15%) exclusively used nurses as intervention delivery personnel. One used physicians to deliver services (3%). Four studies (15%) used a combination of personnel, but not exclusively medical professionals. Eight studies (30%) were unclear on the makeup of their intervention delivery personnel. The majority of studies under review (*n* = 19, 70%) have incorporated education components in their interventions including self-care and monitoring, lifestyle modifications, and/or medication administration and adjustment. Seven studies (26%) incorporated interactive approaches, in which patients were not only receiving one-way messaging but also engaged in two-way communication with health professionals. Intervention periods in reviewed studies ranged from 6 weeks to 1 year, with a median length of 6 months.

### Risk of Bias

The risk of bias assessment of the studies is shown in Supplemental Fig. 1. Twenty-two (81%) of the 27 studies reported and described an appropriate method of randomization, but only 7 (26%) reported an adequate allocation concealment process. Ten (37%) of all studies performed blinding for participants and personnel. In all studies, either assessors were blinded or the outcome measurement is not likely to be influenced by the lack of blinding. Twenty-three (85%) of the 27 studies addressed the reasons for incomplete data. Majority of studies (*n* = 26, 96%) included all expected outcomes, including those that were pre-specified. We did not find additional sources of bias across all studies.

Funnel plots (Supplemental Fig. 2a–f) for six outcomes all display mild asymmetry, suggesting the potential for publication bias. However, the results of the fail-safe N tests for each CVD risk factor except weight indicate that a large additional number of studies would have to be added before the loss of statistical significance would occur. This indicates that publication bias may not be a serious issue in our analysis. Moreover, the trim-and-fill method [54] shows an imputed effect size is the same as or very close to the original effect for each outcome, indicating that minor publication bias, if there is any, is not sufficient to fundamentally alter our results (Supplemental Table 2).

### Quantitative Results

**Fig. 2:**
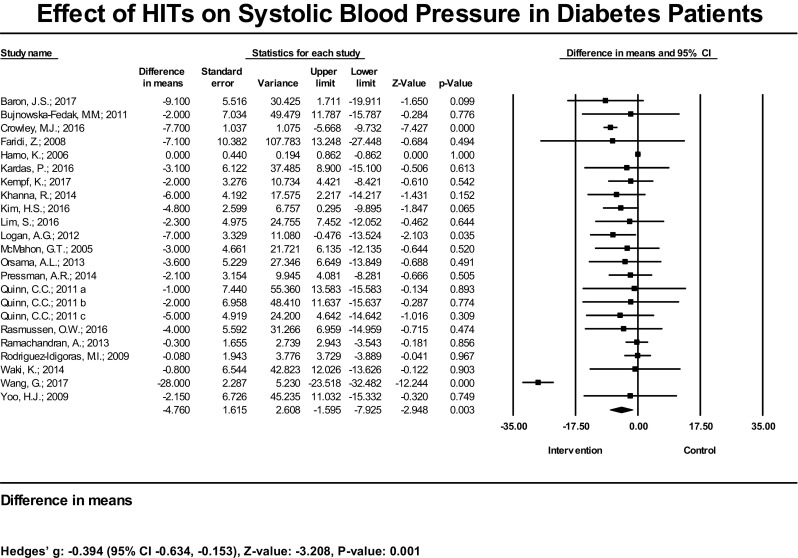
Effect of HIT on systolic blood pressure in patients with T2D—meta-analysis results from 21 RCTs (23 estimates) assessing the effect of HIT on systolic blood pressure. Squares indicate a study-specific mean difference of the outcome; horizontal lines indicate 95% CIs; diamond indicates the summary mean difference estimate with its 95% CI. Under the figure, bias-adjusted effect size (Hedges’ g) and its 95% CIs are also provided

With respects to BP reduction, 21 studies (23 SBP estimates) assessed the effect of HIT on SBP. Among these, 20 studies found a statistically non-significant reduction in SBP, and 3 studies showed statistically significant SBP reductions. The mean reduction in SBP resulting from HIT across studies was statistically significant at − 4.76 mmHg (95% CI − 7.93, − 1.60 mmHg), *P* < 0.001 (Fig. 2). The bias-adjusted effect size (Hedges’ g) was − 0.39 (95% CI − 0.63, − 0.15), *P* = 0.001. In the subset analysis where we explicitly examined trials comparing HIT plus standard care interventions vs. standard care controls, we found a significant mean reduction at − 5.18 mmHg (95% CI − 7.94, − 2.41), *P* < 0.001, and a significant bias-adjusted effect size (Hedges’ g) of −0.58 (95% CI − 1.05, − 0.10), *P* = 0.019 (Table 1).

**Table 1:** Effect of HIT on CVD risk factors from RCTs comparing HIT + standard care interventions vs. standard care controls—results from subset meta-analysis^1^

	SBP (95% CI) *n*=6^2^	*P*	DBP (95% CI) *n*=5^2^	*P*	HDL (95% CI) *n*=5^2^	*P*	LDL (95% CI) *n*=4^2^	*P*	TG (95% CI) *n*=4^2^	*P*
Difference in means	− 5.17 (− 7.94, − 2.41)	< 0.001	− 4.09 (− 6.02, − 2.16)	< 0.001	2.04 (− 1.02, 5.10)	0.192	− 8.90 (− 15.85, − 1.96)	0.012	− 15.35 (− 34.76, 4.06)	0.121
Hedges’ g	− 0.58 (− 1.05, − 0.10)	0.019	− 0.69 (− 1.32, − 0.06)	0.031	0.16 (− 0.02, 0.35)	0.087	− 0.30 (− 0.46, − 0.14)	< 0.001	− 0.26 (− 0.45, − 0.06)	0.009

^1^These are subset analyses, where we performed separate meta-analyses for five CVD risk factors separately using data from RCTs explicitly comparing HIT + standard care interventions vs. standard care controls. Both difference in means and Hedges’ g are presented with their 95% CI

^2^Number of studies in subset analyses are 6 for SBP, 5 for DBP, 5 for HDL, 4 for LDL, and 4 for TG, respectively

**Fig. 3:**
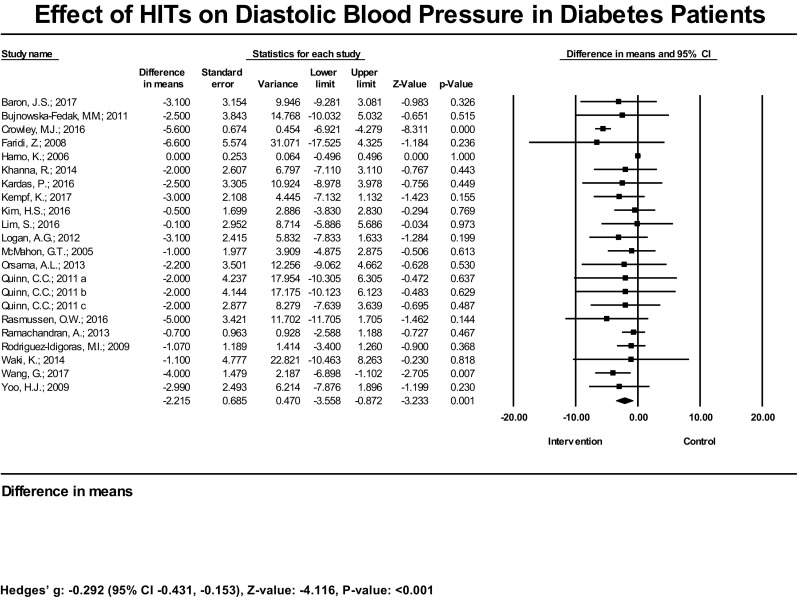
Effect of HIT on diastolic blood pressure in diabetes patients—meta-analysis results from 20 RCTs (22 estimates) assessing the effect of HIT on diastolic blood pressure

Twenty studies (22 estimates) examined the effect of HIT on DBP. Two out of the 20 studies had a statistically significant reduction in DBP; 18 did not find a significant reduction. The mean reduction of DBP was significant at − 2.22 mmHg (95% CI − 3.56, − 0.87 mmHg) (Fig. 3). The bias-adjusted effect size was also significant [Hedges’ g = − 0.29 (95% CI − 0.43, − 0.15), *P* < 0.001]. In the subset analysis, we found the mean reduction was significant across trials comparing HIT plus standard care interventions vs. standard care controls [− 4.09 mmHg (95% CI − 6.02, − 2.16 mmHg), *P* < 0.001] and the bias-adjusted effect size was significant as well [Hedges’ g = − 0.69 (95% CI − 1.32, − 0.06), *P* = 0.031] (Table 1). There was substantial heterogeneity in the effect of interventions on SBP (*I*^2^ = 88%) and DBP (*I*^2^ = 71%) (Supplemental Table 3).

**Fig. 4:**
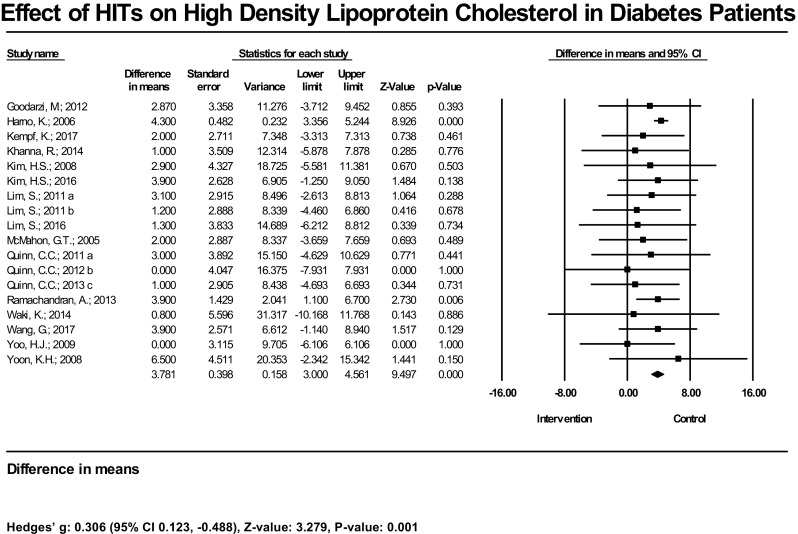
Effect of HIT on high-density lipoprotein cholesterol in diabetes patients—meta-analysis results from 15 RCTs (18 estimates) assessing the effect of HIT on high-density lipoprotein cholesterol

**Fig. 5:**
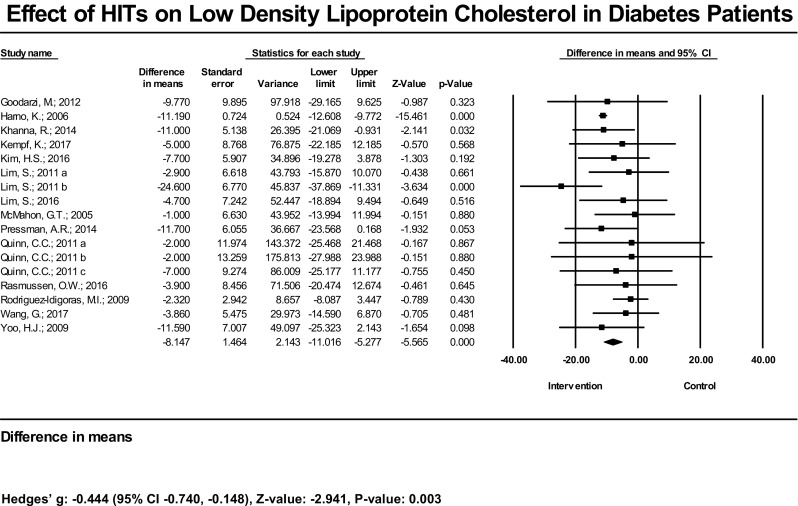
Effect of HIT on low-density lipoprotein cholesterol in diabetes patients—meta-analysis results from 14 RCTs (17 estimates) assessing effect of HIT on low-density lipoprotein cholesterol

**Fig. 6:**
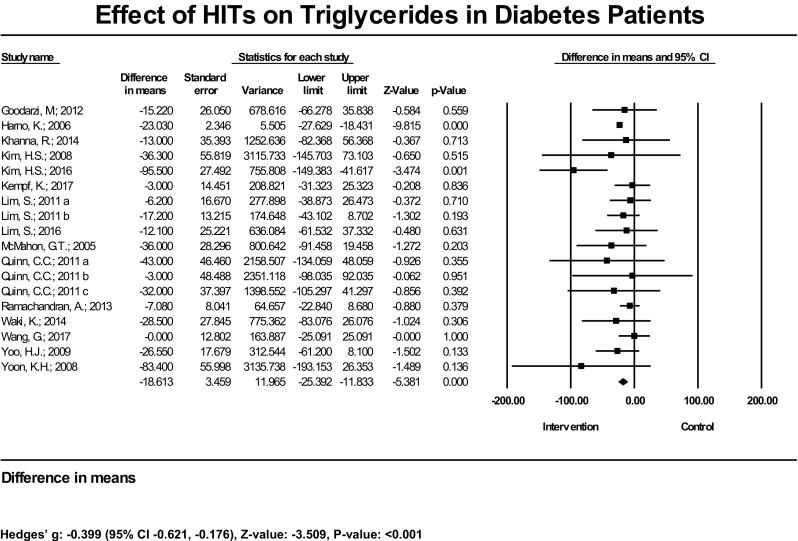
Effect of HIT on triglycerides in diabetes patients—meta-analysis results from 15 RCTs (18 estimates) assessing the effect of HIT on triglycerides

In terms of results in lipid management, among 15 studies (18 estimates) with measured HDL, 2 had statistically significant increases in this outcome; 13 had null findings. The mean increase of HDL was significant at 3.78 mg/dL (95% CI 3.00, 4.56 mg/dL), *P* < 0.001, and the bias-adjusted effect size also showed a significant result [Hedges’ g = 0.31 (95% CI 0.12–0.49), *P* = 0.001] (Fig. 4). In the subset analysis, the mean increase of HDL was not significant (2.04 mg/dL, 95% CI − 1.02, 5.10, *P* = 0.192). The bias-adjusted effect size [Hedges’ g = 0.16 (95% CI − 0.02, 0.35), *P* = 0.087] was not significant as well (Table 1). Three out of 14 studies (17 estimates) had significant reductions in LDL; 11 did not find significant reduction. The mean decrease of LDL was significant at − 8.2 mg/dL (95% CI − 5.3, − 11.0 mg/dL) (Fig. 5). The bias-adjusted effect size was also significant [Hedges’ g = − 0.44 (95%CI − 0.74, − 0.15), *P* = 0.003]. In the subset analysis, the common effect on LDL reduction was significant. The difference in means was − 8.15 mg/dL (95% CI − 15.85, − 1.96), *P* = 0.012, and the Hedges’ g was − 0.30 (95% CI − 0.467, − 0.14), *P* < 0.001 (Table 1). Two out of 15 studies (18 estimates) had a statistically significant reduction in TG; 13 had no significant findings. The mean TG reduction was significant at − 18.6 mg/dL (95% CI − 11.8, − 25.4 mg/dL), and Hedges’ g was − 0.40 (95% CI − 0.63, − 0.18), *P* < 0.001 (Fig. 6). In the subset analysis, we found the mean reduction of TG resulting from HIT was not significant [− 15.35 mg/dL (95% CI − 34.76, 4.06), *P* = 0.121]; however, the bias-adjusted effect size was significant [Hedges’ g = − 0.26 (95% CI − 0.45, − 0.06), *P* = 0.009) (Table 1).

**Fig. 7:**
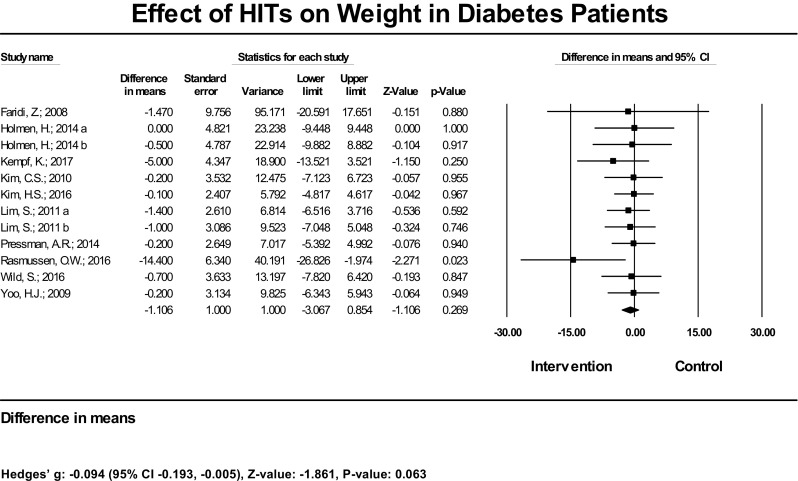
Effect of HIT on weight in diabetes patients—meta-analysis results from 12 RCTs (13 estimates) assessing the effect of HIT on weight

Twelve studies (13 estimates) examined the effect of HIT on weight. However, no study showed a significant effect on the outcome. The mean weight decrease was − 1.10 kg (95% CI − 3.06, 0.85) (Fig. 7). Among reviewed trials that assessed weight outcome, no one exclusively compared HIT plus standard care intervention vs. standard care control, therefore, no subset analysis was performed.

## Discussion

In our meta-analysis evaluation of HIT’s effect in six important CVD risk factors among patients with T2D participating in 22 HIT trials on T2D management, we found significant improvements in SBP, DBP, LDL, HDL, and TG attributed to HIT. There are several likely mechanisms through which HIT affects CVD risk factors. HIT can help motivate patients toward lifestyle changes such as improved diet, exercise, or weight loss [55, 56]. They can also improve adherence to prescribed antihypertensive and lipid-lowering medications [4]. Lastly, HIT may alert patients and/or their care team that risk factors are less than optimal and may need intervention, such as is the case with HIT interventions that help patients and their care teams monitor blood pressure [57].

We were concerned that the measured effect of HIT on CVD risk factors may represent a mixed effect from standard care, including drug-based treatments and lifestyle modifications. To address this concern, we purposefully selected trials that compared CVD risk factors between combined HIT and standard clinical treatments including medication and lifestyle interventions and a standard care alone control group. All these trials centered on testing the effect of HIT but included standard care as supporting components. Our results from this subset analysis showed significant and important effects of HIT on SBP, DBP, LDL, and TG, though not on HDL. These results are in line with what we found from the overall analysis [4].

Reported effects of HIT may not be directly comparable to what is reported in pharmaceutical trials focusing on testing the effect of specific antihypertensive or antihyperlipidemic drugs for patients with significantly elevated CVD risk factors [15, 58]. However, HIT may hold promise in CVD risk management among people with T2D as they appear to achieve equivalent or even greater effect as some lifestyle interventions designed for CVD risk factor management. For example, it has been reported that the loss of 1 kg in body weight can result in a decrease in mean arterial BP of 1 mmHg [58]. A moderate daily sodium restriction (from a daily intake of 200 mmol to 100 mmol) can lead to a reduction in SBP around 5 mmHg and DBP 2–3 mmHg [58]. These results are very similar to the SBP (− 4.76 mmHg) and DBP (− 2.22 mmHg) reduction, respectively, associated with HIT in our study.

In terms of LDL reduction, an increase in soluble fiber consumption led to 2.2 mg/dL reduction in LDL, an increase in phytosterol consumption led to 13 mg/dL reduction in LDL, an increase in nut consumption led to 10.2 mg/dL reduction in LDL, and an increase in daily soy isoflavone consumption led to 5 mg/dL LDL reduction in LDL [59]. The effect of HIT on LDL that we quantified (− 8.2 mg/dL) is within the range seen with these lifestyle modifications.

With respect to HDL, aerobic exercise training resulted in a 2 mg/dL increase in HDL [60], smoking cessation was associated with a 3.8 mg/dL increase [61], and Mediterranean diet led to a 3.8 mg/dL increase [62]. Our estimated effect of HIT on HDL (3.78 mg/dL) was in the range of that seen with these lifestyle interventions. Our comparable results to effective lifestyle interventions indicate a promising role of HIT in CVD factor risk management for patients with T2D. Additionally, HIT has potential in enhancing medication adherence [4], in promoting healthy lifestyles [63–66], and in supporting health risk assessment and monitoring [6], all of which may further aid in overall diabetes management.

We did not find a significant weight reduction associated with HIT among patients with T2D as reported in other studies, especially in lifestyle intervention studies [67]. However, weight loss trials in patients with diabetes that focus on behavioral changes often show weight plateau after 4 to 6 months [68], in part due to decreased energy expenditure [69, 70] and increased calorie retention over time, as well as psychological fatigue [71]. It remains to be seen whether the use of HIT in conjunction in addition to effective pharmaceutical therapies may increase accountability and aid in reaching weight loss goals [4].

In contrast to null findings from two previous meta-analyses that also included examinations of HIT’s effect on CVD risk factors among patients with diabetes [13, 14], our results showed a significant impact of HIT on CVD risk factor management, especially on BP and cholesterol. The null findings reported previously may be due to modest sample sizes included in their CVD risk meta-analysis as well as narrower HIT categories included in the review. Pal et al. [13] only focused on computer-based interventions, and Marcolino et al. [14] focused only on telemedicine. Our study, however, covered a broad spectrum of HIT including mobile communication devices (cell phone, tablet, computers, and PDAs), web-based (web portals, e-mailing), telemedicine, and messaging/SMS.

The heterogeneity observed in BP outcomes in our study may be explained by the wide variety of interventions included. Interventions under review ranged from broad, simple messages providing diabetes management suggestions for patients [72] to more comprehensive interventions permitting timely communication with and instructions from diabetes care managers via phone call, SMS, and telemetry devices [30, 73]. Heterogeneity may also stem from variations in intervention designs, the type of care or services offered to the control groups, differing involvements of health care personnel including different types of personnel, and variations in sample composition (e.g., nationality, age, race/ethnicity).

### Limitations

There are several limitations to the study. First, more than half of the reviewed studies did not provide clear information on blinding to participants and personnel on outcome measurement. Evidence has suggested that the lack of blinding is unlikely to influence an objectively assessed outcome such as BP and lipids [74]. Second, the current review did not include papers published in non-English language or trial registry data. However, we used broad inclusion/exclusion criteria to increase the likelihood of capturing relevant studies to minimalize the publication bias. We included a manual search of reference lists of eligible articles, relevant systematic reviews, and narrative reviews. Third, due to unavailable information about medications that participating patients were taking for CVD risk factor management, we were not able to fully separate the effect of HIT from the possible medications taken in the reviewed trials. We have partially addressed this issue by analyzing the effect on CVD risk factors for HIT plus standard care interventions versus standard care controls in our subset analysis.

## Conclusions

The clinical implication of the favorable impact of HIT on CVD risk factors, especially on BP and cholesterol among patients with T2D, is important because these risk factors are strong predictors of microvascular and macrovascular complications in individuals with T2D. This study suggests that HIT may have a positive impact on the management of BP and lipid levels among patients with diabetes. Quality diabetes care should consider the use of HIT for management of CVD risk factors in diabetes, especially among patients who are not at recommended BP or lipid targets. Future studies should focus on elucidating the adoptability and feasibility of different HIT based strategies for CVD risk factor management among individuals with T2D.

### Funding Sources and Disclaimers

This publication was made possible by Grant Number 1P30DK092950 from the NIDDK, and its contents are solely the responsibility of the authors and do not necessarily represent the official views of the NIDDK.

The findings and conclusions in this article are those of the authors and do not necessarily represent the official position of the Centers for Disease Control and Prevention.

## Electronic Supplementary Material

ESM 1 (DOCX 65 kb)

ESM 2 (PPTX 79 kb)

ESM 3 (XLSX 17 kb)

ESM 4 (DOCX 15 kb)
