# Supplementary material for: Effect of Health Information Technologies on Cardiovascular Risk Factors among Patients with Diabetes
Source: Curr Diab Rep. 2019 Apr 27;19(6):28. doi: 10.1007/s11892-019-1152-3 (PMC6486904; doi:10.1007/s11892-019-1152-3)
Supplement: Supplementary file 1 — (DOCX 65 kb) [file 11892_2019_1152_MOESM1_ESM.docx]

**Supplemental Figure 1. Risk of Bias Assessment.**
