# Supplementary figures and images for: Effect of Health Information Technologies on Cardiovascular Risk Factors among Patients with Diabetes
Source: Curr Diab Rep. 2019 Apr 27;19(6):28. doi: 10.1007/s11892-019-1152-3 (PMC6486904; doi:10.1007/s11892-019-1152-3)

## Slide 1
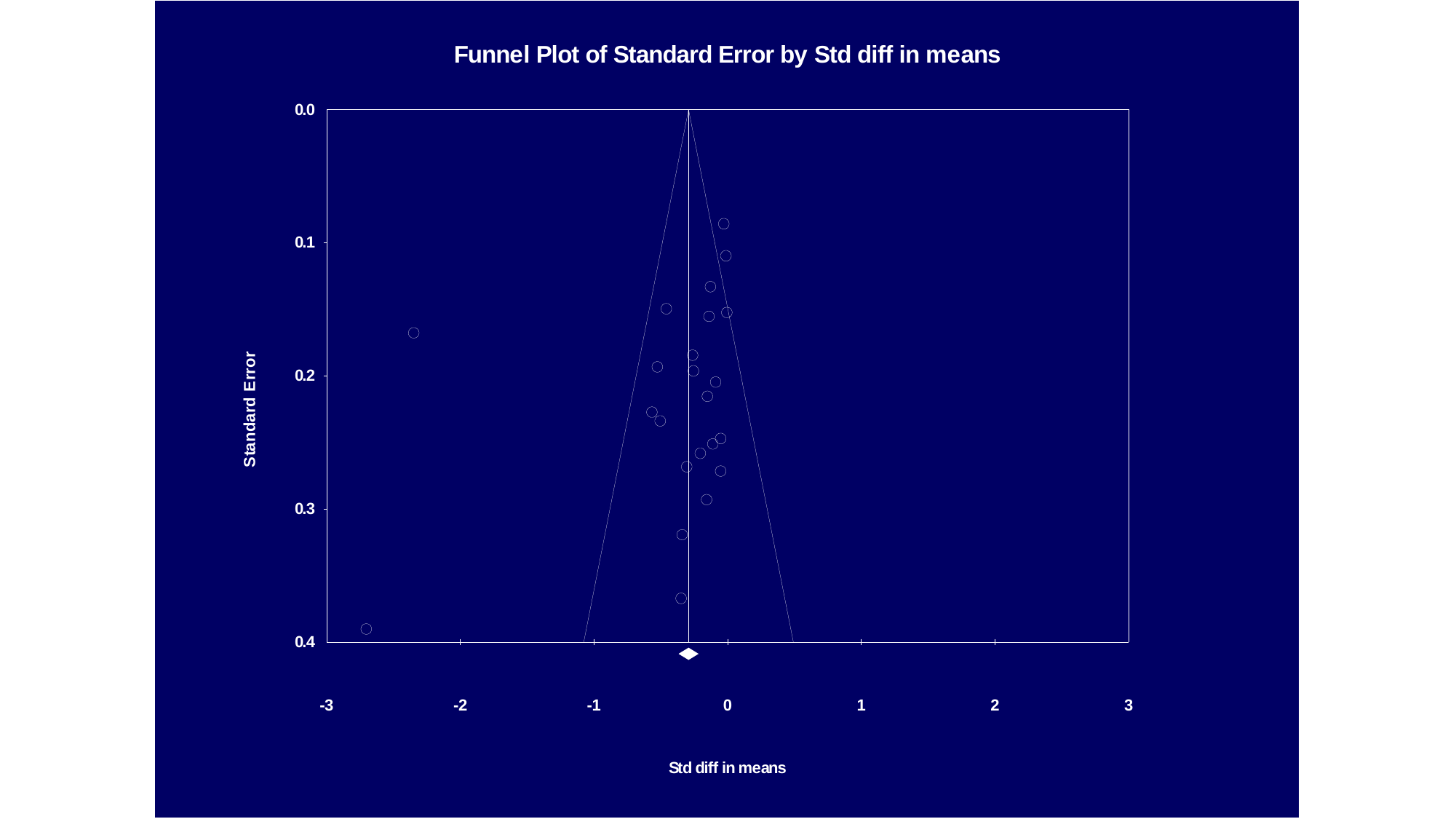

## Slide 2
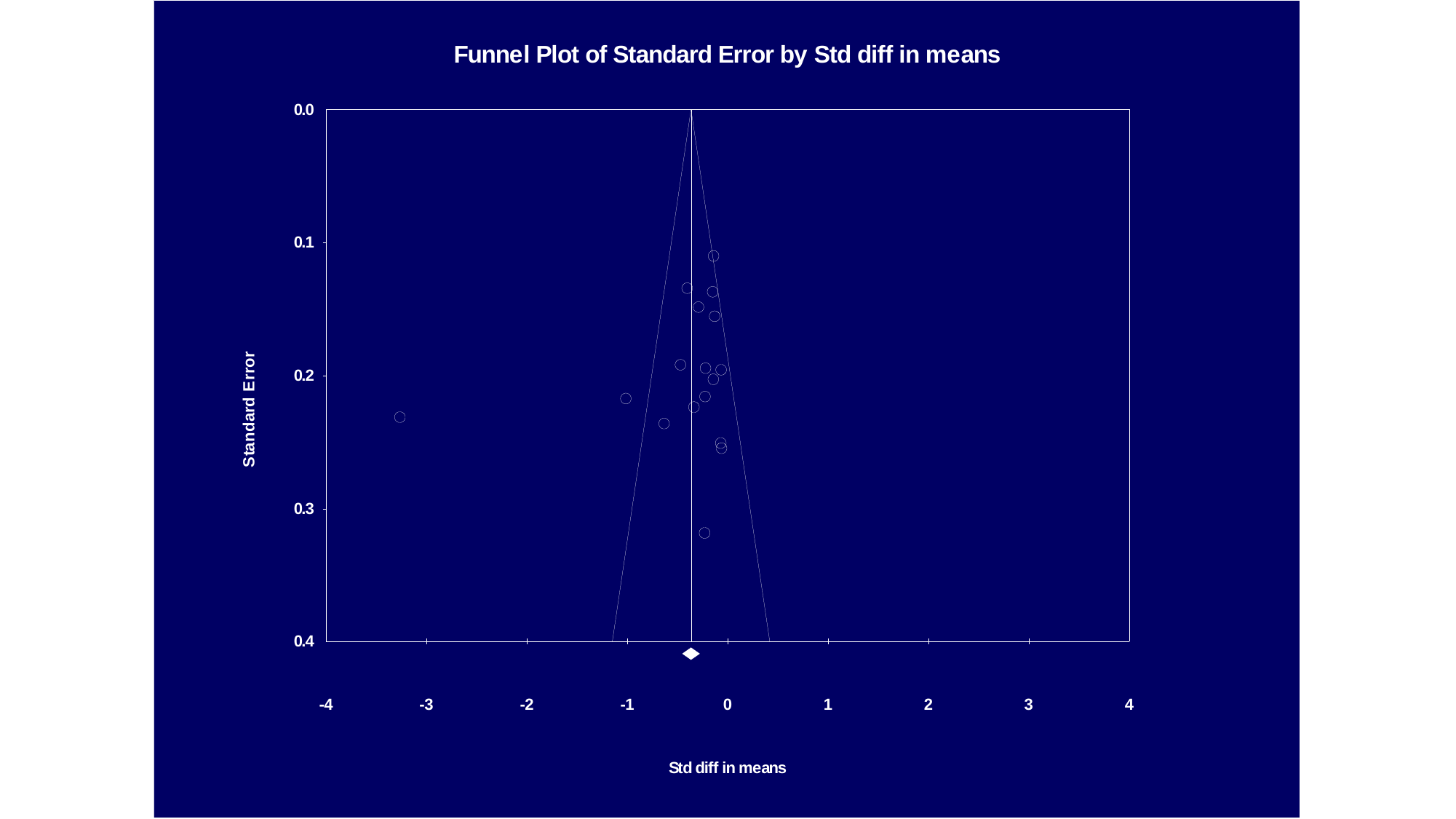

#

## Slide 3
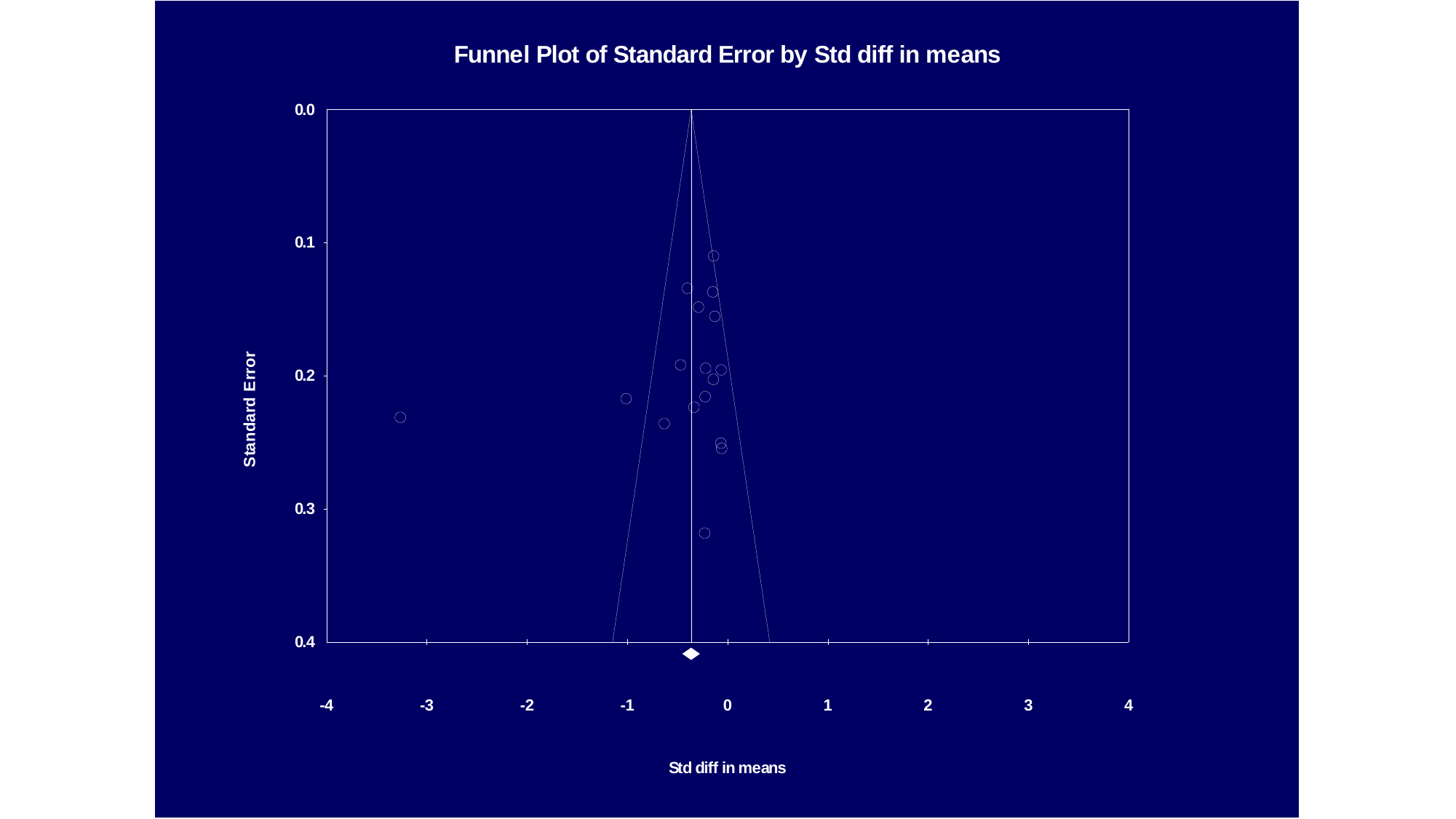

## Slide 4
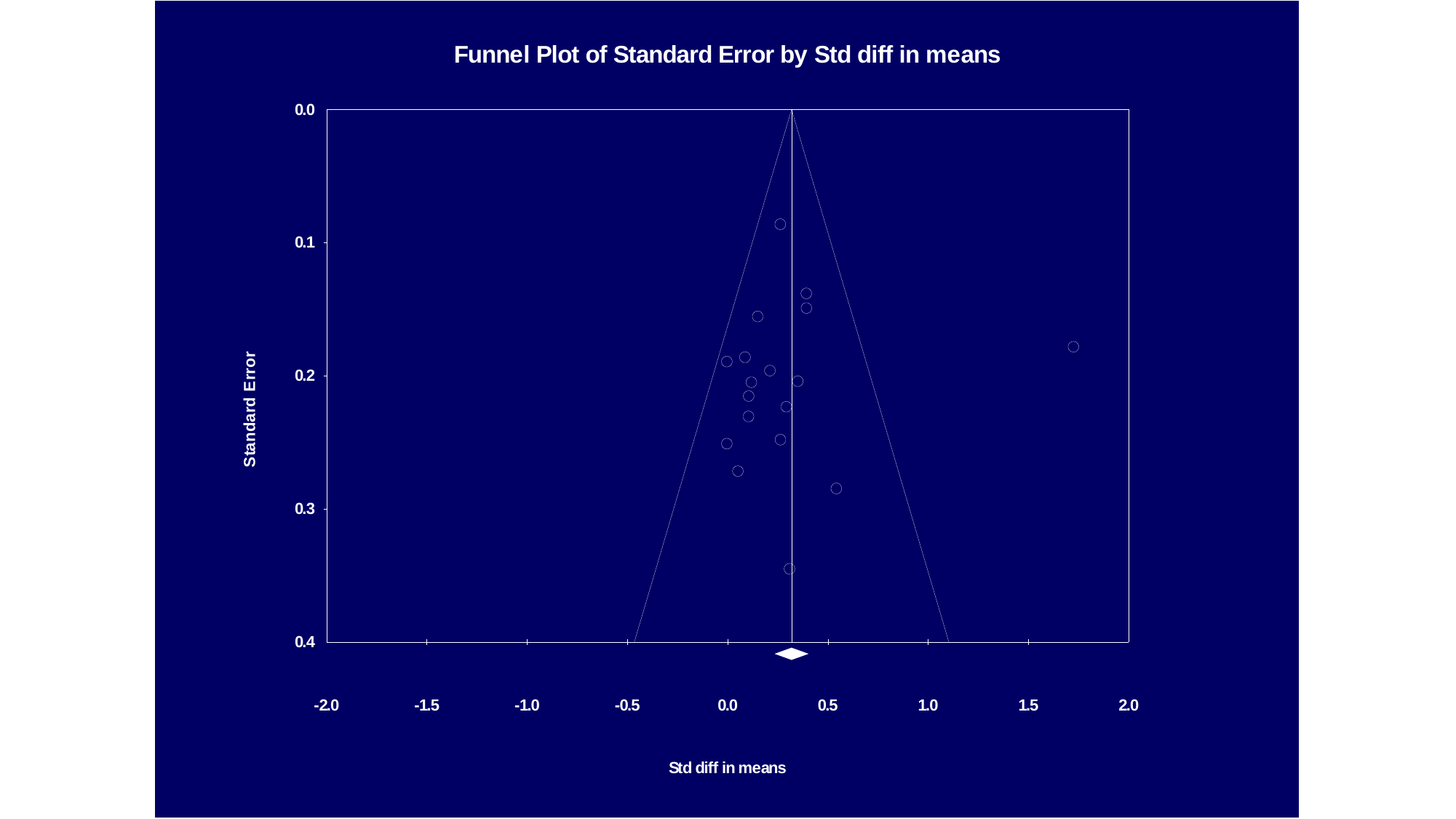

#

## Slide 5
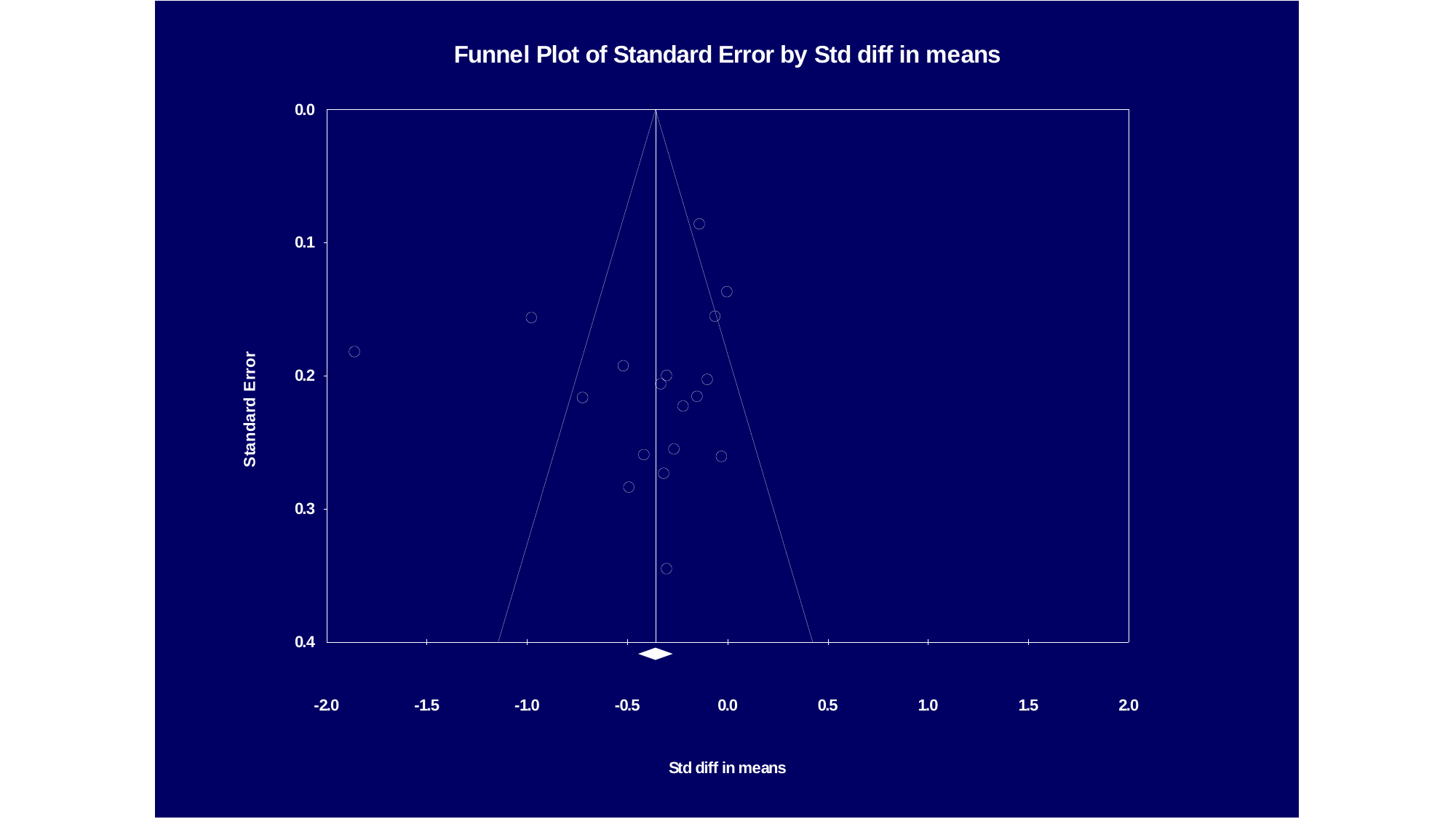

#

## Slide 6
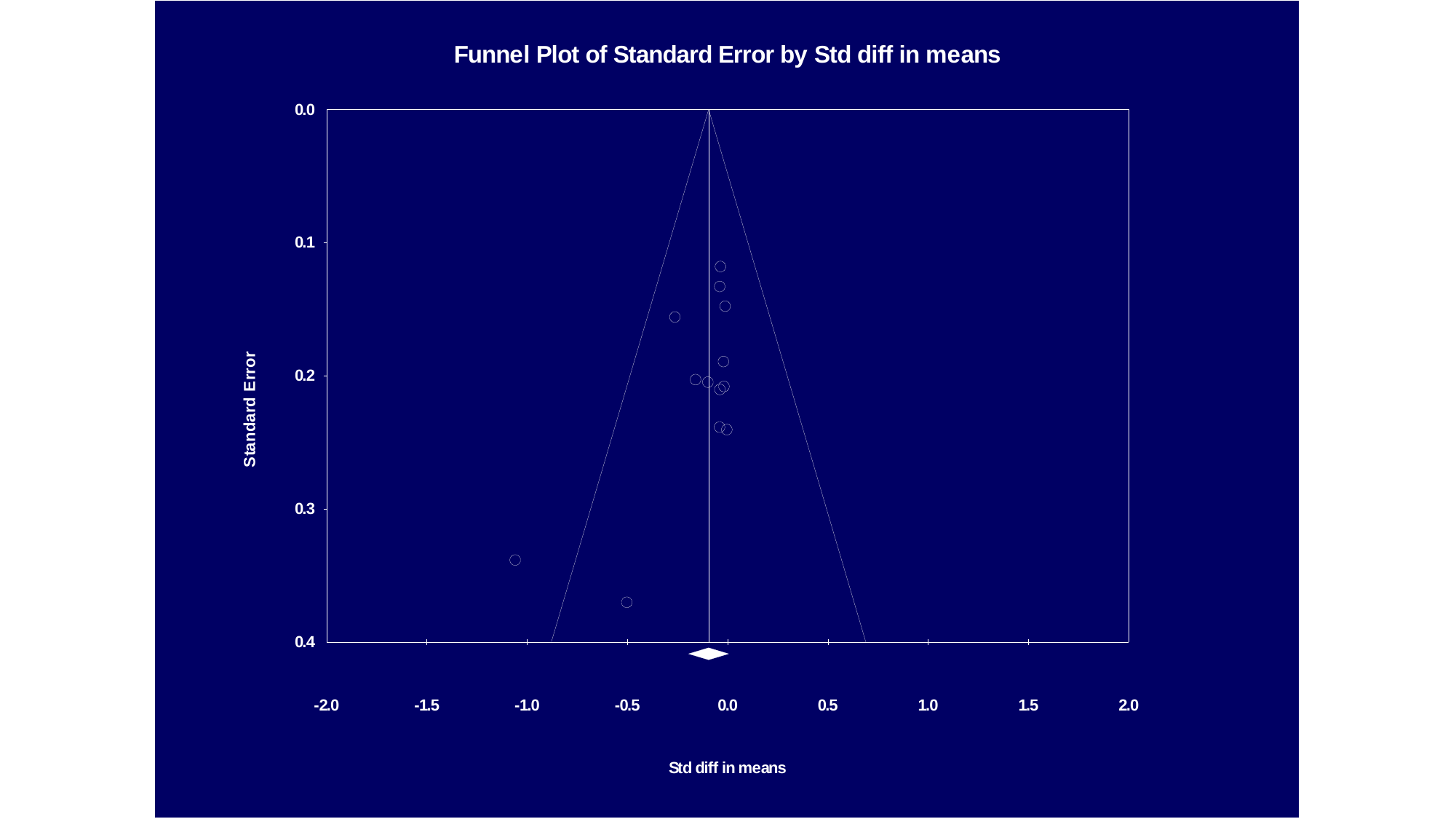

Supplement: Supplementary file 2 — (PPTX 79 kb) [file 11892_2019_1152_MOESM2_ESM.pptx]
