# Supplementary material for: Effect of Health Information Technologies on Cardiovascular Risk Factors among Patients with Diabetes
Source: Curr Diab Rep. 2019 Apr 27;19(6):28. doi: 10.1007/s11892-019-1152-3 (PMC6486904; doi:10.1007/s11892-019-1152-3)
Supplement: Supplementary file 4 — (DOCX 15 kb) [file 11892_2019_1152_MOESM4_ESM.docx]

**Supplemental Table 2. Publication Bias**

| **Outcome** | **Fail-safe N additional studies** | **Trim-and-fill** | **Outcome** |
| --- | --- | --- | --- |
| SBP | 258 | -0.29 (-0.43, -0.15) | -0.29 (-0.43, -0.15) |
| DBP | 439 | -0.59 (-0.82, -0.37) | -0.40 (-0.64, -0.15) |
| HDL | 343 | -0.55 (-0.80, -0.31) | -0.45 (-0.75, -0.31) |
| LDL | 209 | 0.31 (0.12, 0.49) | 0.31 (0.12, 0.49) |
| Triglycerides | 322 | -0.49 (-0.70, -0.29) | -0.40 (-0.63, -0.18) |
| Weight | 6 | -0.10 (-0.20. -0.00) | -0.10 (-0.20, -0.00) |

**Supplemental Table 3. Heterogeneity of HITs Effect on CVD Outcomes (Difference in means)**

| **CVD measurements** | **Heterogeneity** | | |
| --- | --- | --- | --- |
|  | **Q** | **I^2^ %** | **P-value** |
| SBP | 189.1 | 88.3 | 0.000 |
| DBP | 72.4 | 71.0 | 0.000 |
| HDL | 8.0 | 0.0 | 0.968 |
| LDL | 20.8 | 23.0 | 0.188 |
| Triglycerides | 18.6 | 8.8 | 0.349 |
| Weight | 5.7 | 0.0 | 0.89 |
